# Impact of Whey Protein Edible Coating Containing Fish Gelatin Hydrolysates on Physicochemical, Microbial, and Sensory Properties of Chicken Breast Fillets

**DOI:** 10.3390/polym14163371

**Published:** 2022-08-18

**Authors:** Forouzan Sabzipour-Hafshejani, Armin Mirzapour-Kouhdasht, Diako Khodaei, Mohammad Sadegh Taghizadeh, Marco Garcia-Vaquero

**Affiliations:** 1 Department of Natural Resources and Environmental Engineering, School of Agriculture, Shiraz University, Shiraz 71441-65186, Iran; 2 School of Agriculture and Food Science, University College Dublin, Belfield, Dublin 4, D04 V1W8 Dublin, Ireland; 3 Department of Sport, Exercise and Nutrition, Atlantic Technological University, ATU Galway, H91 T8NW Galway, Ireland; 4 Institute of Biotechnology, School of Agriculture, Shiraz University, Shiraz 71441-65186, Iran

**Keywords:** whey protein, fish gelatin hydrolysates, edible biopolymers, active coating, poultry

## Abstract

This study aims to research the impact of coatings containing whey protein (WP), fish gelatin hydrolysates (FGH), and both compounds together (WP + FGH) on the shelf-life of chicken breast fillets over the course of 16 days of cold storage (4 °C, 4-day intervals), as assessed by their physicochemical, microbiological, and sensory properties. Overall, cooking loss, pH value, total volatile base nitrogen, free fatty acids, peroxide value, and thiobarbituric acid reactive substances increased with storage time in all samples. WP + FGH coated samples had significantly lower variation in all these parameters over the time of storage compared to other coated samples (WP and FGH), while these parameters increased greatly in control (uncoated) samples. WP + FGH coating also resulted in reduced bacterial counts of total mesophilic, aerobic psychrotrophic, and lactic acid bacteria compared to other coated and uncoated samples. The sensory evaluation revealed no differences in the panelists’ overall acceptance at day 0 of storage between samples. The samples were considered “non-acceptable” by day 8 of storage; however, WP + FGH coated samples maintained an overall higher acceptability score for the sensory attributes evaluated by the panelists. Overall, this study shows the potential of WP + FGH coatings for prolonging the shelf-life of chicken breast fillets.

## 1. Introduction

The use of edible films and coatings in food packaging is a relatively novel approach to increase the shelf-life of food products. Edible coatings form a protective layer around the food products, helping to reduce moisture loss, respiration rate, and textural deterioration of the foods, as well as reducing the loss of volatile compounds that contribute to the sensory properties of the products [1]. Moreover, the application of coatings in the food surface can create a physical barrier against moisture and gases, contributing to an overall reduction in microbial growth on the surface of the coated foods [2]. Polysaccharide-based or protein-based edible coatings due to their neutral colors and flavors are the most commonly used biopolymers for the generation of edible films and coatings [3]. These biopolymers can be also used to deliver different functional compounds or bioactives, such as peptides, nanoparticles, essential oils, nutrients; as well as other living organisms, such as probiotics, helping to promote or complement the healthy attributes of coated foods [4]. The chemical nature of the antimicrobial agents and/or food in which those compounds are applied as well as other processing conditions (food storage and handling conditions), can all contribute to the antimicrobial efficiency of this coating strategy [5]. Thereby, protein-based films and coatings are reported to have stronger mechanical and gas/moisture barrier properties compared to polysaccharide-based ones. Whey protein-based edible films and coatings are highly transparent, odorless, and have low permeation to oxygen [6]. However, these coatings are negatively affected by humidity and, thus, this factor limits the current applications of these polymers in foods [7].

Additionally, protein hydrolysis involves the breakdown of proteins into peptides and free amino acids that are easier and faster to absorb by the digestive system compared to their parent proteins [8] and exert multiple biological activities. Fish has higher protein content compared to most terrestrial animals [9], and fish proteins have satiating effects and are easy to digest [10]. Fish is one of the most traded food commodities across the world, generating a huge amount of by-products rich in protein that are currently considered as protein losses within this industry [11]. The enzymatic hydrolysis of fish by-products for the generation of protein hydrolysates offers an additional benefit for these industries as these products can be exploited as pharmaceuticals and functional foods [12,13]. Bioactive peptides derived from fish have been frequently described as having additional health benefits when used as food including antihypertensive, anti-inflammatory, immunomodulatory, neuroprotective, and antioxidant effects [14,15]. Peptides obtained from hydrolysis of fish gelatin and collagen have a greater variety of bioavailability and bioactivity—including antioxidant, antimicrobial, angiotensin-converting enzyme (ACE-inhibitory), antihyperglycemic, anti-Alzheimer, and neuroprotective activities—compared to those of raw collagen [5,16].

Chicken breast fillets are prone to microbiological deterioration as the meat composition, high in protein and moisture, offers a suitable substrate for the development of microbes. Additionally, the lipid contents of meat and the oxidation of these compounds under aerobic conditions also contribute to lower meat quality during storage. All these microbial and chemical changes will result in a decrease in the products’ shelf-life, as they will be not suitable or safe for human consumption, as well as financial losses for the food manufacturers. Preventing or delaying the deterioration of chicken breast fillets during refrigeration will be extremely beneficial, and the use of biopolymer coatings can contribute greatly to this purpose [17].

The aim of this study was to investigate the effects of edible coatings, made of WP and/or FGH, on the shelf-life of chicken breast fillets following 16 days of storage under refrigeration conditions. Moreover, the effects of the three coatings (WP, FGH, and WP + FGH) generated in this study on the cooking loss, pH, free fatty acids (FFAs), thiobarbituric acid reactive substances (TBARS), total volatile basic nitrogen (TVB-N), microbial, and sensorial properties of the coated fresh chicken breast fillets were examined and compared to those of uncoated samples.

## 2. Materials and Methods

### 2.1. Biological Materials and Reagents

Fresh chicken breast fillets were purchased from a local market (Shiraz, Iran), immediately transferred to the laboratory in polystyrene bags, and stored in cold boxes containing ice cubes. Barred mackerel (*Scomberomorus commerson*) skins were purchased from a local market (Shiraz, Iran). Alcalase (406.80 U/mg), was purchased from the National Institute of Genetic Engineering and Biotechnology (Tehran, Iran). Actinidin (175.28 U/mg) was obtained from the Institute of Biotechnology, Shiraz University, Shiraz, Iran. Unflavored whey protein concentrate (75% protein, 4.5% carbohydrates, and 5% fat) was purchased from Holland & Barrett (Dublin, Ireland). All other chemicals and reagents were of analytical grade and purchased from Sigma-Aldrich, Inc. (St. Louis, MO, USA).

### 2.2. Gelatin Production and Hydrolysis 

Fish skin gelatin preparation and hydrolysis were performed as described in Mirzapour-Kouhdasht et al. [18] and Mirzapour-Kouhdasht et al. [19] and 1.5% calcium hydroxide solution (pH 6) was used as pretreatment with a 1:10 *w*/*v* ratio at room temperature for 6 h. The extraction of gelatin was performed on pre-treated samples using distilled water at 50 °C for 4 h. Samples were centrifuged (6000× *g*, 20 min) and the supernatants were collected and freeze-dried (IFD-5012, Dena Vacuum, Iran). 

Gelatin extracts were re-dissolved in water (2.5%, *w*/*v*) and subsequently hydrolyzed using alcalase followed by actinidin enzyme, both at a concentration of 1:100 (*w*/*w*), in optimal conditions for alcalase (55 °C, pH 8.5) and actinidin (37 °C, pH 7.5) for 3 h. The hydrolysis was performed as a sequential process, first with alcalase and followed by actinidin. The hydrolysis process was stopped in a water bath at 90 °C for 15 min. The samples were centrifuged (8000× *g*, 20 min) and the supernatants were freeze-dried, vacuum-packed, and stored at −20 °C for further laboratory procedures.

### 2.3. Coating Process

All coating solutions were prepared at room temperature using a magnetic stirrer [20]. WP coating solutions consisted of an aqueous solution of whey protein concentrate (4%, *w*/*v*), FGH was prepared as 1 mg/mL of fish gelatin hydrolysates in distilled water, and WP + FGH prepared by mixing WP and FGH with a ratio of 1:1. Full chicken breast fillets were immersed twice for 2 min in each coating solution. The fillets were then drained using polystyrene nets and refrigerated (4 °C, 30 min) to complete the coating process. Control samples were not submerged in any coating solution. Coated and control samples were stored in polyethylene bags and kept at 4 °C until further testing at days 0, 4, 8, 12, and 16.

### 2.4. Proximate Composition Analyses

All proximate composition analyses of chicken breast fillets were performed following the methods as described by the Association of Official Agricultural Chemists (AOAC) procedures [21]. Briefly, the moisture content of the samples was evaluated by weighing 5 g of fresh ground samples, oven-drying the samples (110 ± 2 °C, 6 h) and re-weighing the dried samples to estimate the moisture content. The ash contents were determined by incinerating the samples using a muffle furnace (525 °C, 6 h). The protein contents of the samples were estimated by the Kjeldahl method. Briefly, samples (1 g) were digested in the presence of catalyst tablets using 30 mL of sulfuric acid to determine their protein content. The samples were heated for 30 min at 220 °C, then for another 120 min at 420 °C, to begin the digestive process. In a desiccator, samples were then cooled to room temperature before being distilled with an auto-Kjeldahl apparatus (BUCHI Labortechnik AG, Flawil, Switzerland). A conversion factor of 6.25 was used to determine the protein content. The lipid content of 1 g of samples was determined by the Soxhlet extraction technique using 100 mL of hexane for 6 h at 68 °C. 

### 2.5. Physicochemical Properties of Chicken Breast Fillets

#### 2.5.1. Cooking Loss

The cooking loss of chicken breast fillets was calculated by weighing samples after cooking in a water bath (80 °C, 1 h) and the results expressed as the percentage of weight reduction relative to the initial weight of the samples following the method as described by Barbanti and Pasquini [22].

#### 2.5.2. pH Measurements

The pH of the samples was measured following the method as described by Mirzapour-Kouhdasht and Moosavi-Nasab [20]. Briefly, suspensions of samples were prepared in distilled water (10%, *w*/*v*) at room temperature and the pH of the solutions measured using a pH meter (Laboratory pH meter, PH 110, Nashua, NH, USA).

#### 2.5.3. Free Fatty Acid (FFA) Contents

FFA contents of the samples were determined following the method described by Horwitz and Latimer [23]. Briefly, 5 g of samples were cold pressed in the presence of 25 mL of ethanol containing 1% phenolphthalein. The lipids extracted from the samples were titrated with 0.1 N NaOH until they reached a stable pink color. The following equation was used to calculate the FFA content in terms of oleic acid:

FFA content (%) = (N × V × 282)/(W × 1000) × 100 (1)

where 282 is the molecular weight of oleic acid; N, the normality of the NaOH solution (mol/L); V, the volume of NaOH used (mL); and W, the sample weight (g).

#### 2.5.4. Peroxide Values (PVs)

PVs were calculated in the lipid extract of the samples using the AOAC 965.33 method [24]. Lipids extracted from the sample were weighed in a 250 mL Erlenmeyer flask, and mixed thoroughly with 25 mL of an acetic acid:chloroform solution (3:2, *v*/*v*). One milliliter of a saturated potassium iodide solution (made by adding ≥144 g KI in 100 mL water at 25 °C) was added to the mixtures under constant stirring for 1 min, followed by the addition of 0.5 mL of starch solution (1%, *w*/*v*), and 30 mL of distilled water. The mixtures were subsequently titrated with 0.01 N sodium thiosulfate. Blank or control samples were determined by following the same procedures without adding the lipid samples. PVs of the samples were estimated using the following equation:

PV content (meqO_2_·kg^−1^ lipid) = ((S−B) × N × 1000)/W (2)

where S, B, N, and W represent the volume consumed during titration (mL), volume consumed in the evaluation of the blank (mL), normality of the sodium thiosulfate solution, and sample weight (g), respectively. PV results were expressed as meqO_2_·kg^−1^ lipid.

#### 2.5.5. Thiobarbituric Acid Reactive Substances (TBARS)

TBARS analysis was performed as detailed by Song et al. [25]. Briefly, 5 g of samples or control (malondialdehyde (MDA)) were dispersed in 20 mL of thiobarbituric acid solution (0.25 M HCl containing 0.375% thiobarbituric acid and 15% trichloroacetic acid). The generated dispersion was boiled in water for 10 min, cooled, and centrifuged (3600× *g*, 20 min, 25 °C). The absorbance of the reactions was read using a spectrophotometer (Shimadzu UV1650 PC, Kyoto, Japan) at 532 nm.

#### 2.5.6. Total Volatile Basic Nitrogen (TVB-N)

TVB-N determinations were performed following previously established methodologies [26]. Briefly, 10 g of samples were minced and distilled in a vessel containing 2% boric acid with methyl red. The samples were then titrated with 0.1 N sulfuric acid until reaching a purple color. TVB-N values were calculated by multiplying the volume of sulfuric acid consumed (UNIT) by a 14 factor that represents the mg N per 100 g of chicken breast fillet [26].

### 2.6. Microbial Analyses

Microbiological assays were conducted as previously described in Mirzapour-Kouhdasht and Moosavi-Nasab [20]. Serial dilutions of samples were prepared by the pour plate method on plate count agar (PCA). The determination of multiple bacteria was performed by changing the incubation conditions of the plates. Mesophilic aerobic bacteria were determined by incubating the plates at 37 °C for 48 h; psychrotrophic bacteria required an incubation of 7 °C for 10 days; and lactic acid bacteria required the use of plates with de Man, Rogosa, and Sharpe (MRS) agar culture medium and incubation in anaerobic conditions for 5 days at 25 °C.

### 2.7. Sensory Properties

The acceptance of samples was assessed by a 10-member panel of semi-trained panelists [27]. The sensory evaluation included an evaluation of the appearance, color, odor, and elasticity/rigidity of the samples. A five-point descriptive hedonic scale was utilized, with 5 indicating intense liking and 1 indicating extreme dislike. The minimum limit of acceptability was set at a score of 3.

### 2.8. Statistical Analyses

All statistical analyses were performed using SPSS (v. 27, SPSS Inc., Chicago, IL, USA). ANOVA tests with Tukey post hoc tests were carried out. In all cases, the criterion for statistical significance was *p* < 0.05.

## 3. Results and Discussion

### 3.1. Physicochemical Changes in Chicken Breast Fillets

#### 3.1.1. Cooking Loss

**Figure 1:**
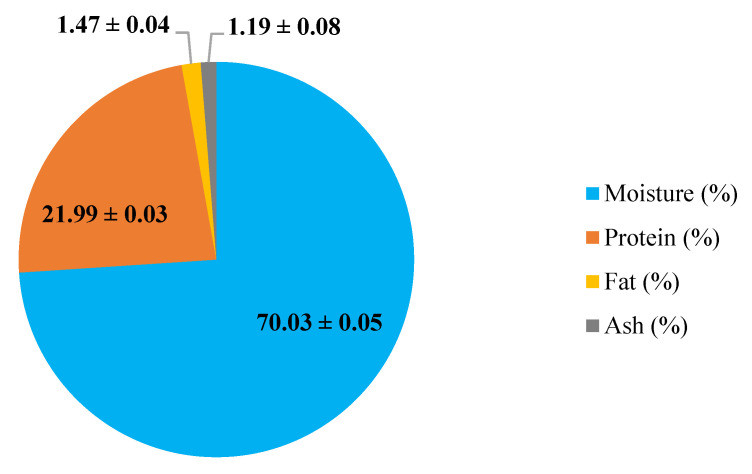
Proximate composition of chicken breast fillets. Results are expressed as average ± standard deviation (*n* = 3).

Cooking loss is an important quality parameter in meat products as it is related to the juiciness of food which influences the consumers’ perception of the final product [28]. Table 1 shows the cooking loss of coated (WP, FGH, and WP + FGH) and uncoated chicken breast fillets during 16 days of storage at 4 °C. With an increased storage time, the differences in cooking loss of coated and uncoated samples became statistically significant (*p* < 0.05). Lee et al. [29] also reported cooking loss in chicken breast fillets of approximately 2.14%, with these values being increased over the storage period of the samples. Uncoated chicken breast fillets had the highest cooking losses, with values 2.85 times higher at 16 days of storage compared to those day 0. There were no significant differences in cooking loss between fillets with different coating treatments (WP and FGH) during 8 days of storage, with cooking loss values increasing by 1.55–1.64-fold after 8 days of storage compared to the values of the samples at day 0. WP + FGH samples exhibited the lowest cooking loss (approximately 47% less) compared to other coated and uncoated samples on days 12 and 16 of storage. The chicken breast fillets in this study were composed of 70% water, 22% protein, 1.5% fat, and 1.2% ash (Figure 1). The evaporation or dripping of water and fats is the main reason for cooking loss [30]. As the fat contents of the fillets is really limited, it can be concluded that the major cause of cooking loss in this study can be mainly attributed to the evaporation or dripping of water from chicken breast fillets. Utilizing edible coatings slows down weight loss during the storage of these food products by lowering the moisture content on the surface and minimizing evaporation [1]. Similar to these results, Küçüközet and Uslu [30] also reported that the use of sodium caseinate coatings reduced the cooking loss in chicken breast fillets with values ranging from 24.93–28.65% in all coated samples, lower than those of uncoated samples (35.27%).

#### 3.1.2. pH Values

**Figure 2:**
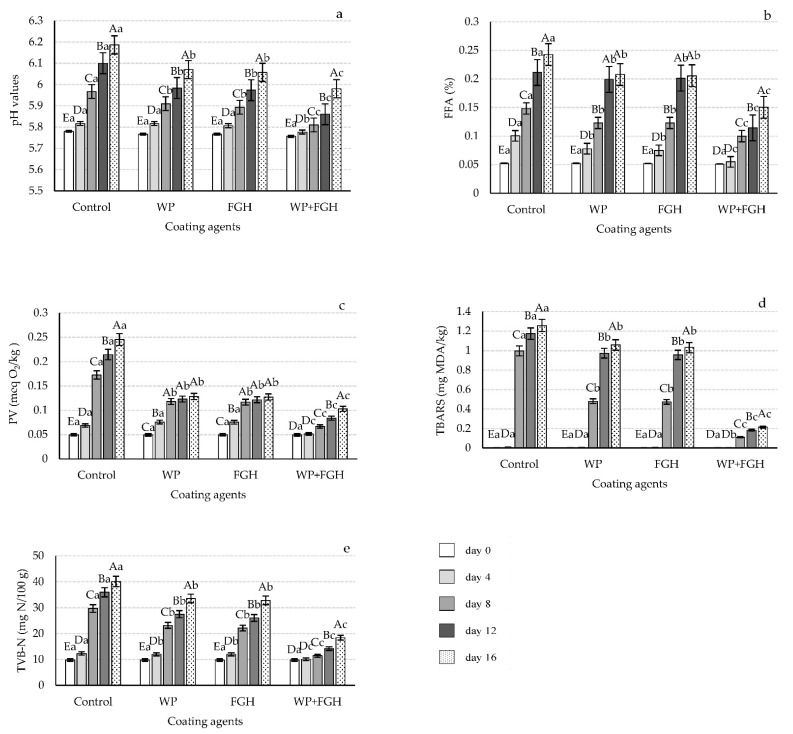
Changes in (**a**) pH value, (**b**) free fatty acid (FFA) contents, (**c**) peroxide value (PV), (**d**) thiobarbituric acid reactive substances (TBARS), and (**e**) total volatile basic nitrogen (TVB-N) of chicken breast fillets coated with whey protein (WP), fish gelatin hydrolysates (FGH), and a combination of both (WP + FGH) during cold storage at 4 °C. Data are represented as mean ± standard deviation (*n* = 3). Different uppercase letters represent statistically significant differences (*p* < 0.05) between various days of storage within samples receiving the same coating treatment; different lowercase letters indicate statistically significant differences (*p* < 0.05) of samples receiving the different coating treatments within the same storage time.

The changes in pH values of different chicken breast fillet samples during the storage period are represented in Figure 2a. The pH values of all chicken breast samples were at their lowest (approximately 5.75) on day zero of storage and no differences can be appreciated between coated and uncoated samples. With the increase in storage time, the pH value for all samples increased. On day 8 of storage, control samples had the highest pH values, followed by FGH and WP samples. Although no significant differences were appreciated between WP and FGH from day 8 onwards, samples coated with WP + FGH exhibited a significantly lower pH value compared to any other sample analyzed, maintaining a pH value of 5.98 on day 16.

The increase in pH values during cold storage in chicken breast fillet samples can be attributed to the microbial growth on the surface of the samples, causing the accumulation of biogenic amines [31]. By inhibiting microbial development on the surface of chicken breast fillets, edible coatings using WP can contribute to delaying the increase in pH of the samples. Moreover, due to its antioxidant and antimicrobial properties, FGH will also slow down the increase in pH during the storage time. Similarly to these results, Mirzapour-Kouhdasht and Moosavi-Nasab [20] also reported that the pH of coated shrimps, with gelatin hydrolysates from *Scomberomorus commerson* skin alone or added to commercial bovine gelatin, was significantly lower compared to that of uncoated samples during 12 days of cold storage under refrigeration conditions. Yıldırım-Yalçın et al. [32] also reported that the pH of coated chicken breast fillets was lower than that of uncoated samples during the 8 days of storage. According to Abdelhedi et al. [33], adding halfbeak (*Hemiramphus far*) skin gelatin hydrolysates to gelatin coatings causes the pores of these microstructures to be filled, reducing the quantity of oxygen and carbon dioxide transferred from the environment to the surface of the food products. 

#### 3.1.3. FFA

The levels of FFA are related to the quality of fat and oils in food products, with high levels of FFA leading to the development of off-odor products [34]. As shown in Figure 2b, FFA values in all samples were at their lowest on day zero, increasing in all the samples during the storage period. Control samples had the highest levels of FFA from day 4 of storage onwards. Using WP and FGH coatings reduced the FFA levels in chicken breast samples compared to control, without any significant differences amongst them. However, both these coatings had increased FFA with storage time following a similar pattern to that of the control samples, although lower in value. Samples coated with WP + FGH had the lowest FFA levels at all time intervals analyzed, with FFA always being lower compared to control, WP, and FGH samples at all time intervals after day 0.

FFA levels in samples are expected to increase during storage time of the samples as the enzymatic hydrolysis of esterified lipids leads to an increase in FFA during refrigerated storage [35]. The low FFA in coated samples might be attributed to the antioxidant activity of both WP and FGH coatings decreasing the formation of FFA. The physical barrier properties of both WP and other protein hydrolysates can reduce the peroxidation of lipids by decreasing the contact of lipids with oxidizing agents [36]. In a similar study, Kchaou et al. [37] reported that protein hydrolysates generated from cuttlefish skin gelatin films had UV barrier and antioxidant activities which makes them useful for packaging food products. 

#### 3.1.4. PV 

Another metric that describes the primary oxidation of unsaturated fats and oils in samples is the PV. In the current study, all samples had very low PV on day zero, with levels of approximately 0.05 meq O_2_/kg (see Figure 2c). However, the PV of all the samples increased with the storage time, with the rate of this increase being higher in uncoated samples compared to any other coated samples, reaching its maximum levels after 16 days of storage (0.25 meq O_2_/kg). WP and FGH significantly inhibited the increase in PV in the samples, with no significant differences between both coatings in helping to decrease the PV levels. Similarly to the other parameters analyzed, the use of WP + FGH was more efficient than any other coating in reducing the PV that reached a maximum level of 0.10 meq O_2_/kg.

The formation of peroxides in meat products has been previously attributed to the hydrolytic effect of microbial enzymes present in these products that will lead to increased PV during cold storage [38]. An increase in PV of chicken breast fillet samples during the shelf-life study was also reported by Kamkar et al. [39] and Bazargani-Gilani et al. [40]. Protein hydrolysates and peptides can help in the processes of reducing hydroperoxides, scavenging of free radicals, inactivation of reactive oxygen species, and modifying the physical properties of the product, acting as antioxidants in these food products [41].

#### 3.1.5. TBARS

TBARS is another relevant parameter to be measured when dealing with the lipid oxidation of meat samples as it is representative of the secondary oxidation products in meat, such as aldehydes or ketones [42]. As shown in Figure 2d, the TBARS values for all chicken breast fillet samples were really low and neglectable up to day 4 of storage. However, from day 8 these values significantly increased in all the samples. Uncoated chicken breast fillet samples had the highest TBARS levels on day 16 (1.25 mg MDA/kg), followed by WP and FGH coated samples of 1.03 and 1.05 mg MDA/kg, respectively. The use of edible coatings containing both WP and FGH was an effective strategy reducing the rate of lipid oxidation in the chicken breast fillet samples in the current study. Edible coatings reduce oxygen permeation, helping to slow down the rate of initial oxidation of lipids and the formation of hydroperoxides [42]. Moreover, the presence of FGH on the surface of chicken breast fillets restricts the oxidation of lipids and lowers the TBARS values that remained at levels of 0.1 mg MDA/kg after 16 days of storage. Yıldırım-Yalçın, Sadıkoğlu, and Şeker [32] observed that using maize starch edible coatings containing grape extract slowed down the increase in TBRAS compared to uncoated chicken breast fillets. Kittiphattanabawon et al. [43] also reported low TBARS values in meat products containing high levels of gelatin hydrolysates. 

#### 3.1.6. TVB-N Values

TVB-N relates to the presence of nitrogenous compounds, such as ammonia, dimethyl, and trimethyl amines, and can be used as an indicator of the level of freshness of food products [44]. The microbial activity of bacteria present in foods leads to enzymatic autolysis, as well as lipid and protein oxidation, inducing the degradation of protein and non-nitrogen components of meat [45]. The degradation of proteins over time causes a rise in organic amines, such as TVB-N [46]. On day 0 of the shelf-life study, all chicken breast fillets had TVB-N values of approximately 10 mg N per 100 g of samples, and these values increased in all samples during their storage (see Figure 2e). Control samples had the highest increment rates in TVB-N values, while the use of WP and FGH helped in reducing these levels. In the case of WP + FGH, this coating delayed the increase in TVB-N values until day 4 of storage, and from day 4 a slight increase in the nitrogenous compounds was appreciated, although they were significantly lower compared to other control and coated samples. The likely cause of the rise in TVB-N level in chicken breast fillets during storage is the accumulation of microbial degradation products or the action of internal enzymes. Similar to our results, Farsanipour et al. [47] and Kamkar, Molaee-Aghaee, Khanjari, Akhondzadeh-Basti, Noudoost, Shariatifar, Sani, and Soleimani [39] reported that the TVB-N in control and coated chicken breast samples increased during the shelf-life study at 4 °C. Moreover, the antioxidant properties of bioactive films based on gelatin cuttlefish (*Sepia officinalis*) skin protein hydrolysates were also previously reported by Kchaou, Jridi, Benbettaieb, Debeaufort, and Nasri [37].

### 3.2. Microbial Properties

**Figure 3:**
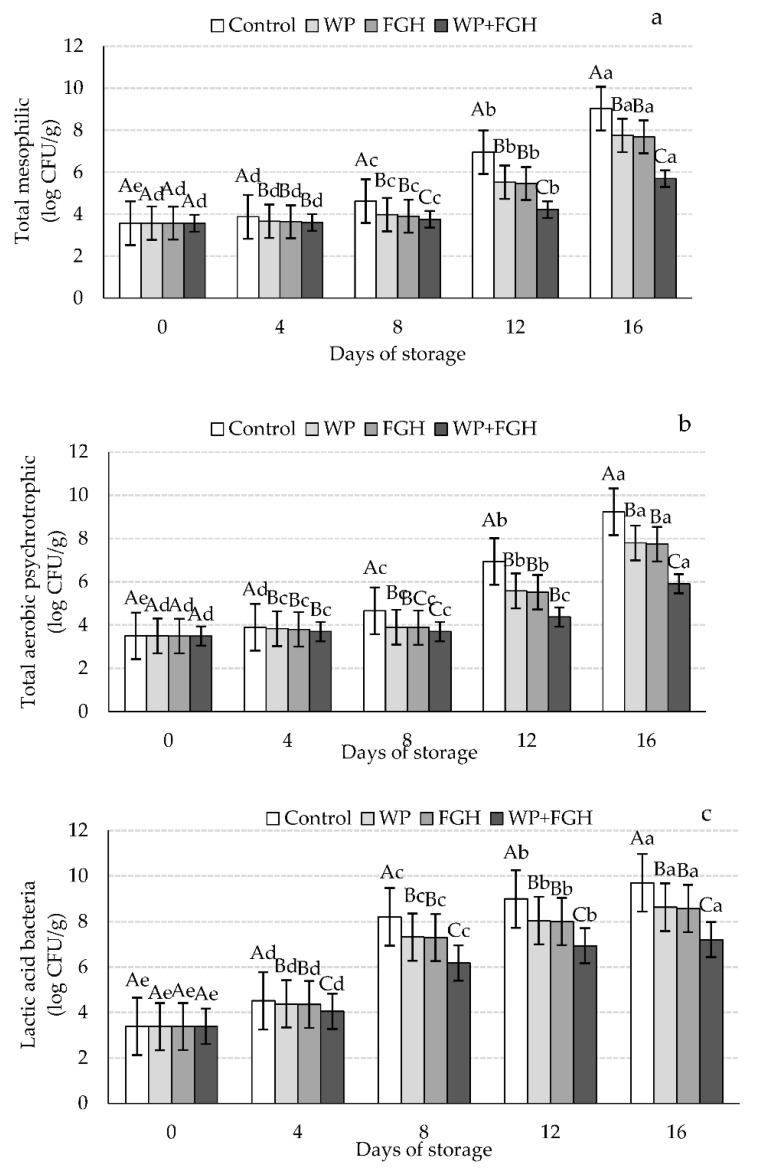
Changes in (**a**) total mesophilic, (**b**) total aerobic psychrotrophic, and (**c**) lactic acid bacterial counts in chicken breast fillets coated with whey protein (WP), fish gelatin hydrolysates (FGH), and a combination of whey protein and fish gelatin hydrolysates (WP + FGH) during cold storage at 4 °C. Data are represented as mean ± standard deviation (*n* = 3). Different uppercase letters represent statistically significant (*p* < 0.05) differences in the microbial counts between days of storage within the same sample type. Different lowercase letters represent statistically significant differences (*p* < 0.05) in microbial counts between different samples within the same storage time.

The effects of different coating treatments on the bacterial counts of (a) total mesophilic, (b) total aerobic psychrotrophic, and (c) lactic acid bacteria (LAB) during the shelf-life period studied are compiled in Figure 3. In the case of total mesophilic bacteria, the initial counts of all the samples were similar, approximately 3.5 log CFU/g, and these levels significantly increased during the storage time. The presence of these bacteria on day 0 is indicative of the initial contamination and microbial load of the product due to slicing and/or packaging processing of the samples [47]. Control uncoated samples had the highest rate of growth in mesophilic bacteria during the 16 days of cold storage, reaching maximum levels of approximately 9.5 log CFU/g sample, higher than the recommended levels of total mesophilic aerobic bacteria (TMAB) in poultry products of 7–8 log CFU/g [48]. Coating samples with WP and FGH delayed the growth of mesophilic bacteria during storage compared to control. Moreover, samples coated with WP + FGH retarded the growth of bacteria up to day 4, when the levels started to increase, reaching a maximum of 5.9 log CFU/g at day 16. The barrier properties of WP, restricting the access of air to TMAB, and also the antioxidant/antimicrobial properties of FGH could be the reason for lower TMAB counts in these samples. Additionally, WPI can stabilize electron-deficient radicals and exhibit antioxidant properties since it contains aromatic and hydrophobic amino acids [49]. The inhibitory properties of whey protein isolate and concentrate coatings have been reported in other studies [50,51]. Yıldırım-Yalçın, Sadıkoğlu, and Şeker [32] reported that the TMAB in uncoated chicken breast samples exceeded the permitted interval after 8 days of storage, while it was still in the acceptable range for coated samples with maize starch coating containing grape extracts. On contrary, other studies reported that WPI coatings had no significant antibacterial properties [52,53].

Similar results were observed in the case of total aerobic bacteria counts, with increased levels of these bacteria in all control and coated samples during the 16 days of storage. The initial aerobic count for chicken breast fillets was ~ 3.8 log CFU/g at day 0 and it increased during the shelf-life study of the samples. The highest levels of aerobic bacteria occurred on day 16, with the highest counts present in the control uncoated samples (9 log CFU/g) and the lowest in WP + FGH samples (approximately 6 log CFU/g). Similarly, Hu et al. [54] reported that using fish skin hydrolysates in chitosan films extended the shelf-life of common carp (*Cyprinus carpio*) stored for 16 days at 4 °C by lowering the growth rate of total viable counts of bacteria. The authors concluded that this resulted in an extended shelf-life of the products, 2 days longer compared to chitosan coated samples and twice of as long when compared to uncoated samples [54].

As shown in Figure 3c, the initial LAB in all samples was ~3 log CFU/g, increasing with the storage time. Control samples had the highest LAB from day 4 onwards, reaching a maximum of 9.70 log CFU/g on day 16 of storage. No significant differences were appreciated between WP and FGH when controlling the number of LAB in the samples. However, WP + FGH had the highest inhibitory effects in LAB, with samples registering the lowest LAB counts (7.21 log CFU/g) compared to any other sample during the entire storage period. Previous studies reported the effects of the use of catfish bone hydrolysates in sausages when inhibiting the growth of microorganisms [55]. The incorporation of fish protein hydrolysates in agar films has also been linked with increased antimicrobial activity of films and extends the shelf-life of flounder (*Paralichthys orbignyanus*) fillets [56]. Balcik Misir and Koral [57] reported that coating bonito (*Sarda sarda*) fillets with fish protein hydrolysates generated from trout by-products had antimicrobial properties against mesophilic, psychrophilic, and coliform bacteria as well as yeast and molds. Mirzapour-Kouhdasht and Moosavi-Nasab [20] reported that using commercial bovine gelatin alone or with FGH increased the shelf-life of shrimps to 6 and 9 days, respectively, compared to 6 days for uncoated samples.

### 3.3. Sensory Evaluation

The total acceptance of chicken breast fillets during the 16 days’ storage period is represented in Table 2. The highest level of acceptance for chicken fillets was observed on day 0 of storage, when all the samples received a score of 5. Thus, using edible WPI or FGH singularly or mixing the two (WPI + FGH) had no negative effect on the perceived sensorial properties of the chicken breast fillets by the panelists. The total acceptance decreased during the storage time for all the samples. TVB-N compounds are toxic and change the quality (color, textural, and taste) of food products, which will directly affect the acceptability of these food products by consumers [58]. The total acceptance for all samples on day 4 was still at acceptable levels (3.66–4) with no significant differences between uncoated or coated samples. The acceptance of all samples after day 8 of storage was drastically reduced, however, samples coated with WP + FGH had higher acceptance (3.66) compared to WP or FGH (2.66 and 3.33) or uncoated samples (2.33) which indicates that this coating significantly helped to retain the sensorial characteristics of the chicken breast fillets.

Other studies have investigated the effects of protein hydrolysates on the sensorial quality parameters of food products. Balcik Misir and Koral [57] reported that using edible coatings containing fish protein hydrolysates improved the overall acceptance of fish fillet samples during the shelf-life study under refrigeration conditions. The sensory criteria for uncoated samples fell below the critical point for sensory evaluation after day 9 of storage, while the coated samples were within the acceptable range up to 12 days of storage. Another study conducted by Parvathy et al. [59] showed that using fish protein hydrolysates from *Euthynnus affinis* for coating iced sardines significantly improved the sensorial properties of the products compared to the control uncoated samples. In another study by Küçüközet and Uslu [30], panelists found that coated wrapping chicken meat roasts were more acceptable than the control samples due mainly to the high tenderness perceived in the coated meat. 

## 4. Conclusions

This study demonstrates the efficiency of WP and FGH coatings, particularly when used combined as WP + FGH, to extend the shelf-life of chicken breast fillets under cold storage conditions, as evaluated by the effects of these coatings in the physicochemical, microbiological, and sensory properties of the food products compared to uncoated samples. Overall, the storage period had a significant effect on the cooking loss, pH, FFA, TVB-N, and TBARS levels of all coated and uncoated samples; however, samples coated with WP + FGH showed the lowest variations in all these parameters for all time periods analyzed in this study. WP + FGH had promising benefits compared to uncoated samples and the use of either WP or FGH individually. The use of edible coatings considerably lowered the microbial counts of mesophilic, aerobic psychrotrophic, and LAB in chicken breast fillets during the storage period analyzed in this study compared to uncoated samples. WP + FGH exhibited the highest benefits when reducing the microbial counts in chicken breast fillets during the 16 days of cold storage. Furthermore, when evaluating the sensory attributes of the samples, the panelists could not distinguish uncoated or coated samples on day zero of storage, indicating that the coating had no effect influencing the sensory attributes of the product. On day 8 of storage, the acceptability of all the samples as evaluated by the panelists was significantly reduced, reaching “non-acceptable” levels. However, samples coated with WP + FGH maintained overall higher acceptability scores compared to those of uncoated or WP and FGH coated samples. Overall, this study demonstrates the promising applications of WP + FGH coatings in the food industry to extend the shelf-life and acceptability of chicken breast fillets, opening possible avenues for the exploitation of these coatings in poultry products, as well as the need for these coating procedures to be evaluated when extending the shelf-life of other food products in further studies. Future studies will be needed on the influence on further physical characteristics (i.e., texture and color) and chemical modifications (antioxidant and biogenic amines) of the products, as well as an optimization of the concentration of biopolymers and their mixtures to ensure optimal food preservation to further support the widespread application of these coatings.

## Figures and Tables

**Table 1:** Cooking loss (%) of chicken breasts fillets coated with whey protein (WP), fish gelatin hydrolysates (FGH), or combination of both (WP + FGH) during prolonged cold storage at 4 °C.

Samples	Days of Storage				
	0	4	8	12	16
Control	20.12 ± 0.10 Ea	26.28 ± 0.08 Da	36.09 ± 0.13 Ca	41.88 ± 0.11 Ba	57.39 ± 0.20 Aa
WP	18.96 ± 0.09 Da	19.97 ± 0.05 Dbc	29.50 ± 0.12 Cbc	36.21 ± 0.08 Bb	50.25 ± 0.23 Ab
FGH	19.28 ± 0.12 Ca	21.21 ± 0.10 Cb	31.63 ± 0.10 Bb	33.74 ± 0.05 Bbc	51.13 ± 0.15 Ab
WP + FGH	18.79 ± 0.10 Ca	19.69 ± 0.11 Cc	27.93 ± 0.10 Bc	32.20 ± 0.02 Bc	47.31 ± 0.14 Ac

Data are expressed as average ± standard deviation (*n* = 3). Different uppercase letters indicate statistical differences (*p* < 0.05) in cooking loss on different days of storage within the same sample type; different lowercase letters indicate statistical differences (*p* < 0.05) in cooking loss between different samples within the same storage time.

**Table 2:** Total acceptance scores of chicken breasts fillets coated with whey protein (WP), fish gelatin hydrolysates (FGH), or a combination of both (WP + FGH) during cold storage at 4 °C. Total acceptance points were considered in a range of 5 (strong like) to 1 (strong dislike). Score of 3 was considered as the limit of acceptability.

Days of Storage	Samples	Total Acceptance Scores
0	Control	5 ± 0.00 Aa
	WP	5 ± 0.00 Aa
	FGH	5 ± 0.00 Aa
	WP + FGH	5 ± 0.00 Aa
4	Control	3.66 ± 0.81 Cb
	WP	4 ± 0.00 Bb
	FGH	4 ± 0.00 Bb
	WP + FGH	4.66 ± 0.00 Aa
8	Control	2.33 ± 0.47 Cc
	WP	3.33 ± 0.00 Bbc
	FGH	2.66 ± 0.00 Bc
	WP + FGH	3.66 ± 0.00 Ab
12	Control	1 ± 0.00 Cd
	WP	2.33 ± 0.00 Bc
	FGH	2.33 ± 0.00 Bc
	WP + FGH	3 ± 0.00 Ac
16	Control	1 ± 0.47 Cd
	WP	1.33 ± 0.00 Bd
	FGH	1.33 ± 0.00 Bd
	WP + FGH	2 ± 0.47 Ad

Data are represented as average ± standard deviation (*n* = 3). Different uppercase letters indicate statistical differences (*p* < 0.05) in total acceptance scores between different samples within the same storage time. Different lowercase letters indicate statistical differences (*p* < 0.05) in cooking loss on different days of storage within the same sample type.

## Data Availability

All data generated or analyzed during this study are included in this published article.

## References

[1] Khodaei D., Hamidi-Esfahani Z. (2019). Influence of bioactive edible coatings loaded with Lactobacillus plantarum on physicochemical properties of fresh strawberries. Postharvest Biol. Technol..

[2] Khodaei D., Hamidi-Esfahani Z., Rahmati E. (2021). Effect of edible coatings on the shelf-life of fresh strawberries: A comparative study using TOPSIS-Shannon entropy method. NFS J..

[3] Khodaei D., Hamidi-Esfahani Z., Lacroix M. (2020). Gelatin and low methoxyl pectin films containing probiotics: Film characterization and cell viability. Food Biosci..

[4] Khodaei D., Oltrogge K., Hamidi-Esfahani Z. (2020). Preparation and characterization of blended edible films manufactured using gelatin, tragacanth gum and, Persian gum. LWT.

[5] Jamróz E., Tkaczewska J., Juszczak L., Zimowska M., Kawecka A., Krzyściak P., Skóra M. (2022). The influence of lingonberry extract on the properties of novel, double-layered biopolymer films based on furcellaran, CMC and a gelatin hydrolysate. Food Hydrocoll..

[6] Galus S., Kadzińska J. (2016). Whey protein edible films modified with almond and walnut oils. Food Hydrocoll..

[7] Shankar S., Khodaei D., Lacroix M. (2021). Effect of chitosan/essential oils/silver nanoparticles composite films packaging and gamma irradiation on shelf life of strawberries. Food Hydrocoll..

[8] Rønnestad I., Kamisaka Y., Conceição L., Morais S., Tonheim S. (2007). Digestive physiology of marine fish larvae: Hormonal control and processing capacity for proteins, peptides and amino acids. Aquaculture.

[9] Egerton S., Culloty S., Whooley J., Stanton C., Ross R.P. (2018). Characterization of protein hydrolysates from blue whiting (*Micromesistius poutassou*) and their application in beverage fortification. Food Chem..

[10] Khora S.S. (2013). Marine fish-derived bioactive peptides and proteins for human therapeutics. Int. J. Pharm. Pharm. Sci..

[11] Harnedy-Rothwell P.A., Khatib N., Sharkey S., Lafferty R.A., Gite S., Whooley J., O’Harte F.P., FitzGerald R.J. (2021). Physicochemical, Nutritional and In Vitro Antidiabetic Characterisation of Blue Whiting (*Micromesistius poutassou*) Protein Hydrolysates. Mar. Drugs.

[12] Kristinsson H.G., Rasco B.A. (2000). Fish Protein Hydrolysates: Production, Biochemical, and Functional Properties. Crit. Rev. Food Sci. Nutr..

[13] de Castro R.J.S., Sato H.H. (2014). Production and biochemical characterization of protease from Aspergillus oryzae: An evaluation of the physical–chemical parameters using agroindustrial wastes as supports. Biocatal. Agric. Biotechnol..

[14] Lees M.J., Carson B.P. (2020). The potential role of fish-derived protein hydrolysates on metabolic health, skeletal muscle mass and function in ageing. Nutrients.

[15] Wangkheirakpam M., Mahanand S., Majumdar R., Sharma S., Hidangmayum D., Netam S. (2019). Fish waste utilization with reference to fish protein hydrolisate—A review. Fish. Technol..

[16] Hajfathalian M., Ghelichi S., García-Moreno P.J., Moltke Sørensen A.-D., Jacobsen C. (2018). Peptides: Production, bioactivity, functionality, and applications. Crit. Rev. Food Sci. Nutr..

[17] Yusof N.L., Mutalib N.-A.A., Nazatul U., Nadrah A., Aziman N., Fouad H., Jawaid M., Ali A., Kian L.K., Sain M. (2021). Efficacy of Biopolymer/Starch Based Antimicrobial Packaging for Chicken Breast Fillets. Foods.

[18] Mirzapour-Kouhdasht A., Lee C.W., Yun H., Eun J.-B. (2021). Structure–function relationship of fermented skate skin gelatin-derived bioactive peptides: A peptidomics approach. Food Sci. Biotechnol..

[19] Mirzapour-Kouhdasht A., Moosavi-Nasab M., Krishnaswamy K., Khalesi M. (2020). Optimization of gelatin production from Barred mackerel by-products: Characterization and hydrolysis using native and commercial proteases. Food Hydrocoll..

[20] Mirzapour-Kouhdasht A., Moosavi-Nasab M. (2020). Shelf-life extension of whole shrimp using an active coating containing fish skin gelatin hydrolysates produced by a natural protease. Food Sci. Nutr..

[21] AOAC (1995). Meat and Meat Products.

[22] Barbanti D., Pasquini M. (2005). Influence of cooking conditions on cooking loss and tenderness of raw and marinated chicken breast meat. LWT-Food Sci. Technol..

[23] Horwitz W., Latimer G. (2000). AOAC Official Methods of Analysis.

[24] Cunniff P. (1997). Aoac peroxide value of oils and fats method 965.33. Official Methods of Analysis of AOAC International.

[25] Song Y., Liu L., Shen H., You J., Luo Y. (2011). Effect of sodium alginate-based edible coating containing different anti-oxidants on quality and shelf life of refrigerated bream (*Megalobrama amblycephala*). Food Control.

[26] Abelti A.L. (2013). Microbiological and chemical changes of Nile Tilapia (*Oreochromis niloticus* L.) fillet during ice storage: Effect of age and sex. Adv. J. Food Sci. Technol..

[27] Kilincceker O., Dogan I.S., Kucukoner E. (2009). Effect of edible coatings on the quality of frozen fish fillets. LWT-Food Sci. Technol..

[28] Gómez I., Ibañez F.C., Beriain M.J. (2019). Physicochemical and sensory properties of sous vide meat and meat analog products marinated and cooked at different temperature-time combinations. Int. J. Food Prop..

[29] Lee Y.S., Saha A., Xiong R., Owens C.M., Meullenet J.F. (2008). Changes in Broiler Breast Fillet Tenderness, Water-Holding Capacity, and Color Attributes during Long-Term Frozen Storage. J. Food Sci..

[30] Küçüközet A.O., Uslu M.K. (2018). Cooking loss, tenderness, and sensory evaluation of chicken meat roasted after wrapping with edible films. Food Sci. Technol. Int..

[31] Hernández-Jover T., Izquierdo-Pulido M., Veciana-Nogués M.T., Vidal-Carou M.C. (1996). Biogenic amine sources in cooked cured shoulder pork. J. Agric. Food Chem..

[32] Yıldırım-Yalçın M., Sadıkoğlu H., Şeker M. (2021). Characterization of edible film based on grape juice and cross-linked maize starch and its effects on the storage quality of chicken breast fillets. LWT.

[33] Abdelhedi O., Jridi M., Nasri R., Mora L., Toldrá F., Nasri M. (2019). Rheological and structural properties of Hemiramphus far skin gelatin: Potential use as an active fish coating agent. Food Hydrocoll..

[34] Thiansilakul Y., Benjakul S., Richards M.P. (2010). Changes in heme proteins and lipids associated with off-odour of seabass (*Lates calcarifer*) and red tilapia (*Oreochromis mossambicus* × *O. niloticus*) during iced storage. Food Chem..

[35] Nirmal N.P., Benjakul S. (2011). Retardation of quality changes of Pacific white shrimp by green tea extract treatment and modified atmosphere packaging during refrigerated storage. Int. J. Food Microbiol..

[36] Loi C.C., Eyres G.T., Birch E.J. (2019). Effect of milk protein composition on physicochemical properties, creaming stability and volatile profile of a protein-stabilised oil-in-water emulsion. Food Res. Int..

[37] Kchaou H., Jridi M., Benbettaieb N., Debeaufort F., Nasri M. (2020). Bioactive films based on cuttlefish (*Sepia officinalis*) skin gelatin incorporated with cuttlefish protein hydrolysates: Physicochemical characterization and antioxidant properties. Food Packag. Shelf Life.

[38] Khezrian A., Shahbazi Y. (2018). Application of nanocompostie chitosan and carboxymethyl cellulose films containing natural preservative compounds in minced camel’s meat. Int. J. Biol. Macromol..

[39] Kamkar A., Molaee-Aghaee E., Khanjari A., Akhondzadeh-Basti A., Noudoost B., Shariatifar N., Sani M.A., Soleimani M. (2021). Nanocomposite active packaging based on chitosan biopolymer loaded with nano-liposomal essential oil: Its characterizations and effects on microbial, and chemical properties of refrigerated chicken breast fillet. Int. J. Food Microbiol..

[40] Bazargani-Gilani B., Aliakbarlu J., Tajik H. (2015). Effect of pomegranate juice dipping and chitosan coating enriched with Zataria multiflora Boiss essential oil on the shelf-life of chicken meat during refrigerated storage. Innov. Food Sci. Emerg. Technol..

[41] Sohaib M., Anjum F.M., Sahar A., Arshad M.S., Rahman U.U., Imran A., Hussain S. (2017). Antioxidant proteins and peptides to enhance the oxidative stability of meat and meat products: A comprehensive review. Int. J. Food Prop..

[42] Mahdavi V., Hosseini S.E., Sharifan A. (2018). Effect of edible chitosan film enriched with anise (*Pimpinella anisum* L.) essential oil on shelf life and quality of the chicken burger. Food Sci. Nutr..

[43] Kittiphattanabawon P., Benjakul S., Visessanguan W., Shahidi F. (2012). Gelatin hydrolysate from blacktip shark skin prepared using papaya latex enzyme: Antioxidant activity and its potential in model systems. Food Chem..

[44] Wells N., Yusufu D., Mills A. (2019). Colourimetric plastic film indicator for the detection of the volatile basic nitrogen compounds associated with fish spoilage. Talanta.

[45] Pellissery A.J., Vinayamohan P.G., Amalaradjou M.A.R., Venkitanarayanan K. (2020). Spoilage bacteria and meat quality. Meat Quality Analysis.

[46] MISIR G.B., Koral S. (2021). The impacts of ultrasound-assisted protein hydrolysate coating on the quality parameters and shelf life of smoked bonito fillets stored at 4 ± 1 °C. Ege J. Fish. Aquat. Sci..

[47] Farsanipour A., Khodanazary A., Hosseini S.M. (2020). Effect of chitosan-whey protein isolated coatings incorporated with tarragon Artemisia dracunculus essential oil on the quality of Scomberoides commersonnianus fillets at refrigerated condition. Int. J. Biol. Macromol..

[48] Senter S.D., Arnold J.W., Chew V. (2000). APC values and volatile compounds formed in commercially processed, raw chicken parts during storage at 4 and 13 °C and under simulated temperature abuse conditions. J. Sci. Food Agric..

[49] Mann B., Kumari A., Kumar R., Sharma R., Prajapati K., Mahboob S., Athira S. (2015). Antioxidant activity of whey protein hydrolysates in milk beverage system. J. Food Sci. Technol..

[50] Yıldız P.O., Yangılar F. (2016). Effects of different whey protein concentrate coating on selected properties of rainbow trout (*Oncorhynchus mykiss*) during cold storage (4 °C). Int. J. Food Prop..

[51] Seifzadeh M. (2014). Effects of whey protein edible coating on bacterial, chemical and sensory characteristics of frozen common Kilka. Iran. J. Fish. Sci..

[52] Brink I., Šipailienė A., Leskauskaitė D. (2019). Antimicrobial properties of chitosan and whey protein films applied on fresh cut turkey pieces. Int. J. Biol. Macromol..

[53] Motalebi A., Hasanzati Rostami A., Khanipour A., Soltani M. (2010). Impacts of Whey Protein Edible Coating on Chemical and Microbial Factors of Gutted Kilka during Frozen Storage. Iran. J. Fish. Sci..

[54] Hu S., Luo Y., Cui J., Lu W., Wang H., You J., Shen H. (2013). Effect of silver carp (Hypophthalmichthys molitrix) muscle hydrolysates and fish skin hydrolysates on the quality of common carp (*Cyprinus carpio*) during 4 °C storage. Int. J. Food Sci. Technol..

[55] Ren X.Q., Ma L.Z., Chu J. Effect of catfish bone hydrolysate on the quality of catfish sausage during ambient temperature (37 °C) storage. Proceedings of the Advanced Materials Research.

[56] Da Rocha M., Alemán A., Romani V.P., López-Caballero M.E., Gómez-Guillén M.C., Montero P., Prentice C. (2018). Effects of agar films incorporated with fish protein hydrolysate or clove essential oil on flounder (*Paralichthys orbignyanus*) fillets shelf-life. Food Hydrocoll..

[57] Balcik Misir G., Koral S. (2019). Effects of Edible Coatings Based on Ultrasound-treated Fish Proteins Hydrolysate in Quality Attributes of Chilled Bonito Fillets. J. Aquat. Food Prod. Technol..

[58] Cao L., Sun G., Zhang C., Liu W., Li J., Wang L. (2019). An intelligent film based on cassia gum containing bromothymol blue-anchored cellulose fibers for real-time detection of meat freshness. J. Agric. Food Chem..

[59] Parvathy U., Nizam K., Zynudheen A., Ninan G., Panda S., Ravishankar C. (2018). Characterization of fish protein hydrolysate from red meat of Euthynnus affinis and its application as an antioxidant in iced sardine. J. Sci. Ind. Res..

